# Supplementary material for: Riboformer: a deep learning framework for predicting context-dependent translation dynamics
Source: Nat Commun. 2024 Mar 5;15:2011. doi: 10.1038/s41467-024-46241-8 (PMC10915169; doi:10.1038/s41467-024-46241-8)
Supplement: Supplementary file 1 — Supplementary Information [file 41467_2024_46241_MOESM1_ESM.pdf]

Supplementary figures

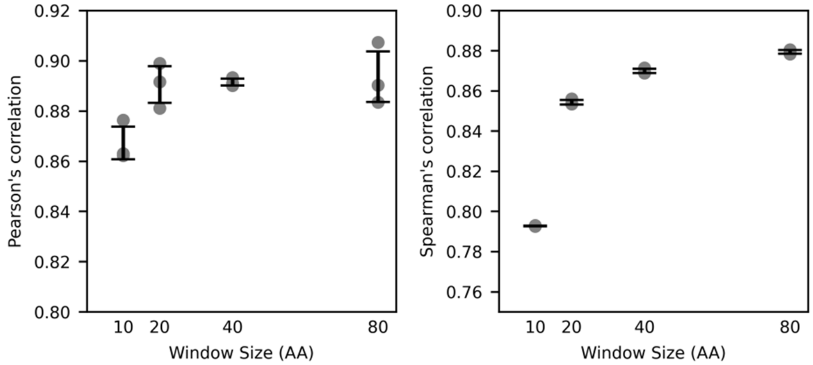

Supplementary Figure 1. Prediction performance of Riboformer in relation to the window size of input sequence. The model was evaluated using 3-fold cross-validation tests on the Mohammad et al. dataset (n = 3). Pearson correlation coefficients (left) and Spearman correlation coefficients (right) between the true and predicted ribosome densities are shown. The error bars represent the standard deviation.

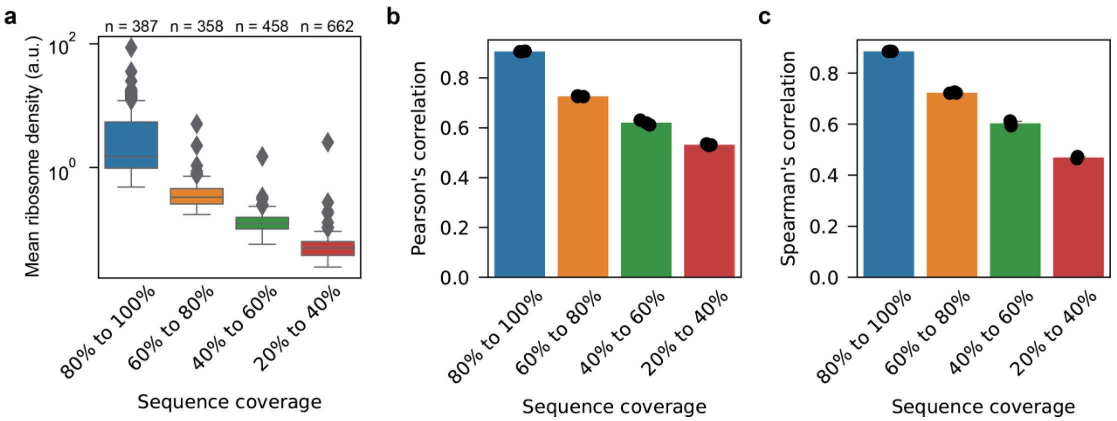

Supplementary Figure 2. **Prediction performance of Riboformer in relation to the sequence coverage of the input data.** The sequence coverage is defined as the proportion of codons with non-zero read counts across the gene coding regions. **a**, average ribosome densities for the genes with different sequence coverage. The number of genes for each group is shown on the top. The central line inside the box represents the median value. The top and bottom borders of the box represent the third (upper) and first (lower) quartiles, respectively. Prediction performance of Riboformer for genes with different sequence coverages in terms of the Pearson correlation coefficient (**b**) and Spearman correlation coefficient (**c**) between true and predicted ribosome densities. The model was evaluated using 3-fold cross-validation tests on the Mohammad et al. dataset (n = 3). The error bars represent the standard deviation.

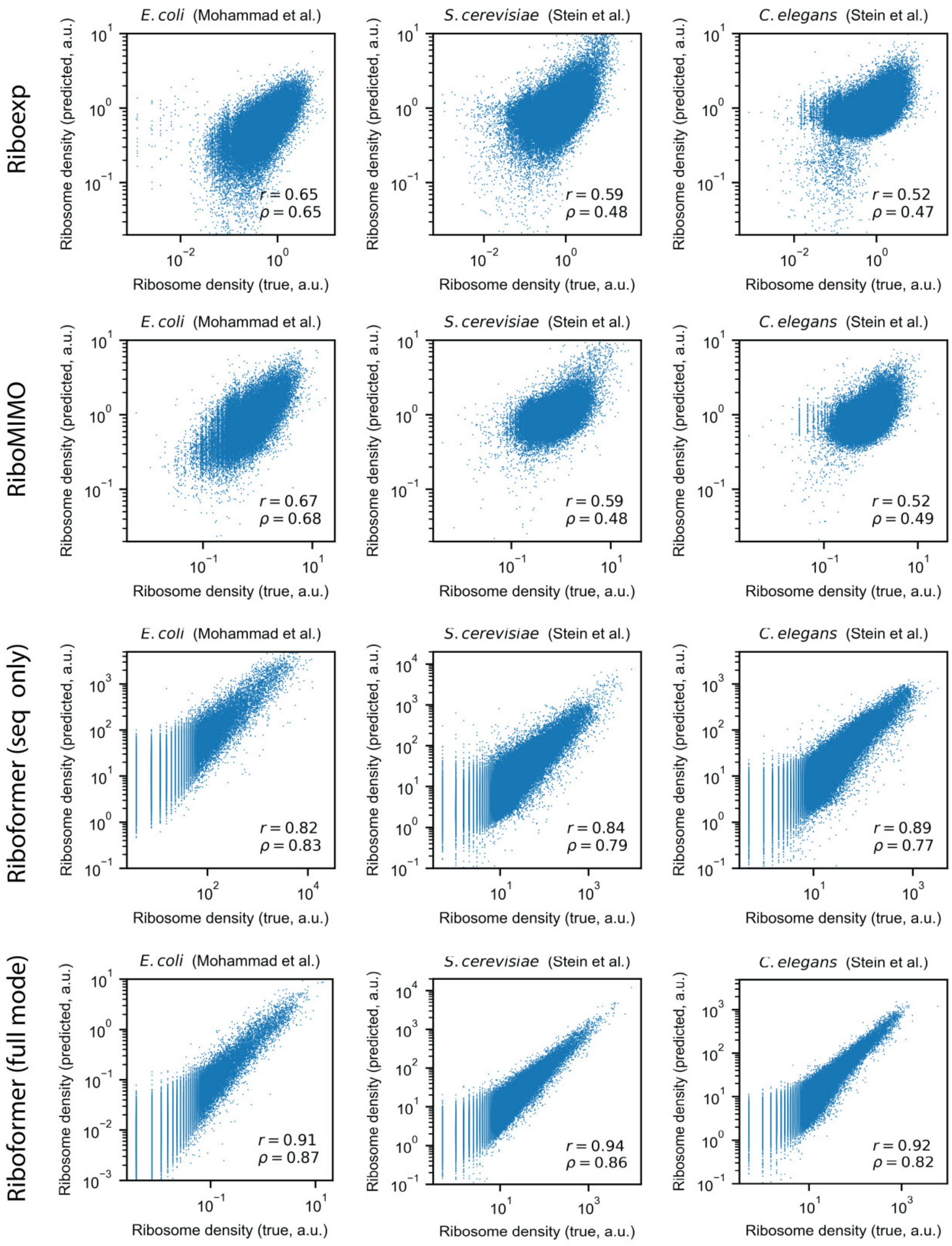

<u>Supplementary Figure 3</u>**. Comparison of the prediction performance of Riboformer with that of different baseline methods across three different species.** Riboformer was tested in two modes: "seq only mode" which uses the coding sequence as the only input, and the "full mode" which takes two inputs including the coding sequence and the reference input. Each dot represents one codon from the independent test datasets. Results from one-fold of the cross-validation tests are shown. The results

from all folds are reported in Supplementary Table 4. *r* and *p* are Pearson and Spearman correlation coefficients between the true and predicted ribosome density for all codons in the test datasets.

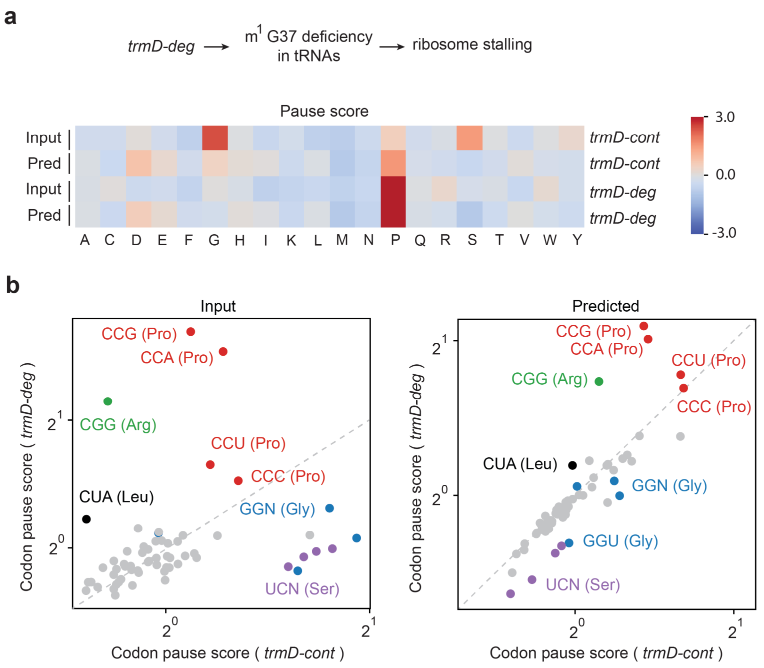

<u>Supplementary Figure 4:</u> Ribosome pausing in *E. coli* cells with m^{1}G37 deficiency. a, mean codon pause scores are shown for the WT (*trmD-cont*) and trmD depletion cells (*trmD-deg*). **b**, pause scores for codons positioned in the ribosomal A site before (left) and after (right) correction for the experimental bias. 61 sense codons are shown individually. Riboformer was used to remove the experimental bias in the ribosome profiles.

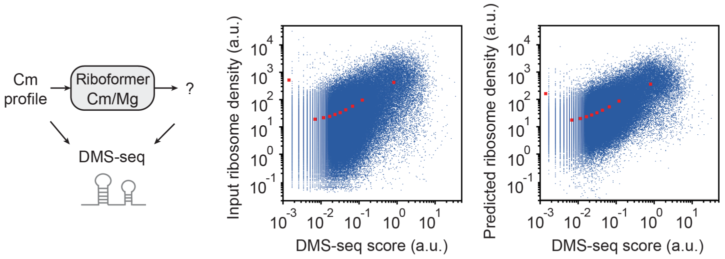

<u>Supplementary Figure 5:</u> Codon level comparison of ribosome density and DMS-seq score. a, the trained Riboformer model was used to correct ribosome profiles in *E. coli*. **b**, codon-level comparison of ribosome density and DMS-seq score before (left, r = 0.26) and after (right, r = 0.32) correction. Each dot is one codon. The binned average of ribosome densities is shown in red squares.

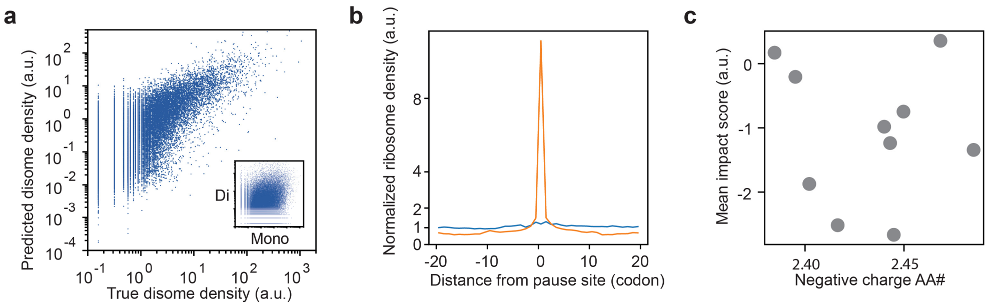

<u>Supplementary Figure 6:</u> Sequence determinants of disome peaks in yeast. a, monosome and disome profiling datasets are used to train the Riboformer model. The predicted disome density and the true disome density in the test dataset is shown (*r* = 0.75). Each dot is a single codon of interest. Inset, comparison of disome density and monosome density (*r* = 0.35). **b,** average ribosome occupancy of the disome profiles around disome peaks (orange) and the average ribosome occupancy in the monosome profiles (blue) are shown, n = 11,079. The monosome and the disome profiles are normalized in the 40-codon window by dividing the mean number of reads across the window. **c**, Comparison between the number of negative charged amino acids and the SIS in yeast (*r* = -0.07). Each dot represents the mean number of the negative charged amino acids in the input sequence and the mean SIS for one cluster (Fig. 2c).

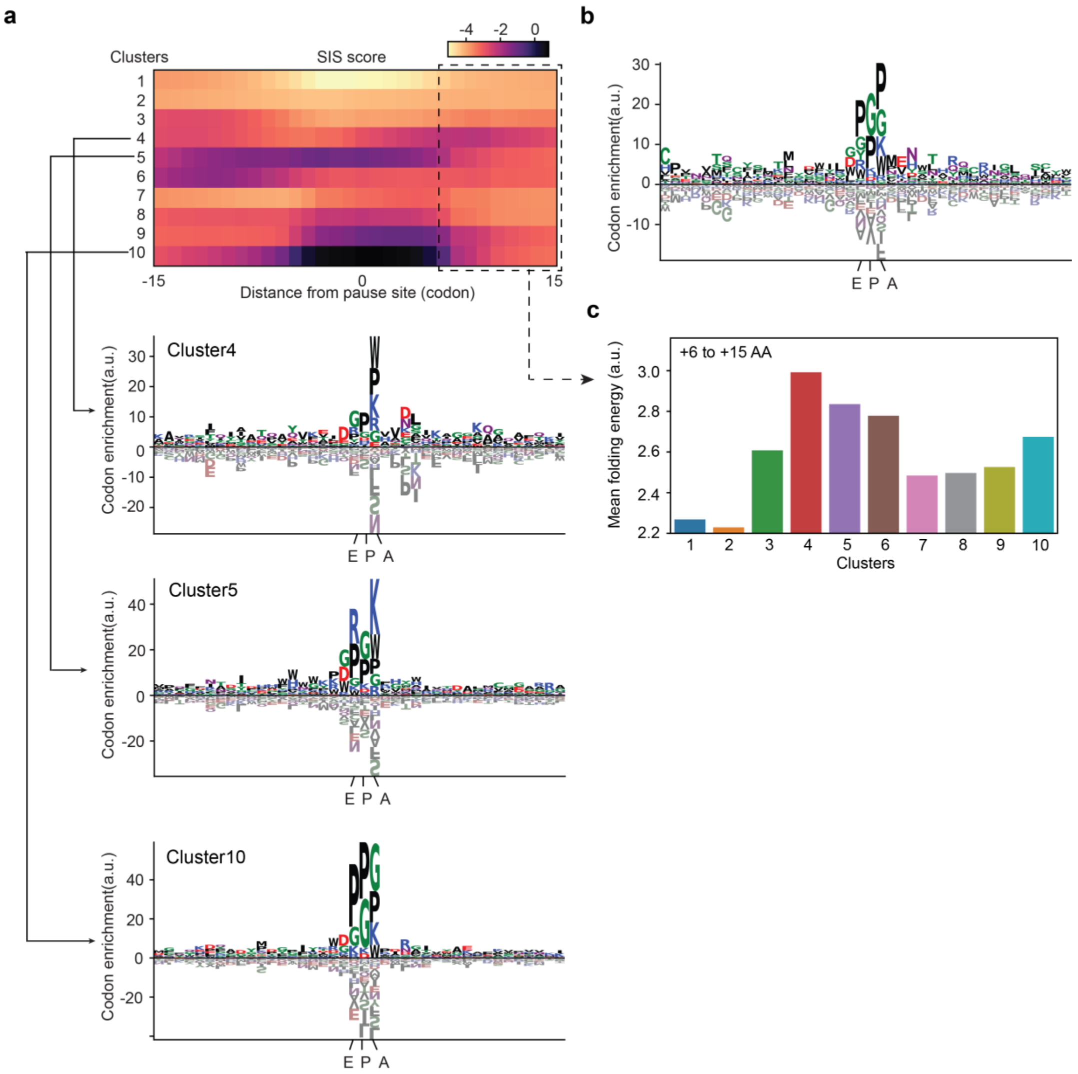

<u>Supplementary Figure 7</u>. SIS analysis of disome formation sites. a, SIS profiles of all the disome peaks are grouped into 10 clusters (top), and codon enrichment profiles for cluster 4 (n = 675), cluster 5 (n = 497), and cluster 10 (n = 272) are shown (bottom). **b**, codon enrichment profile for all disome peaks (n = 11,079). **c**, mean folding energy of the input mRNA sequence for all clusters over a 10 AA window (+6 AA to +15 AA).

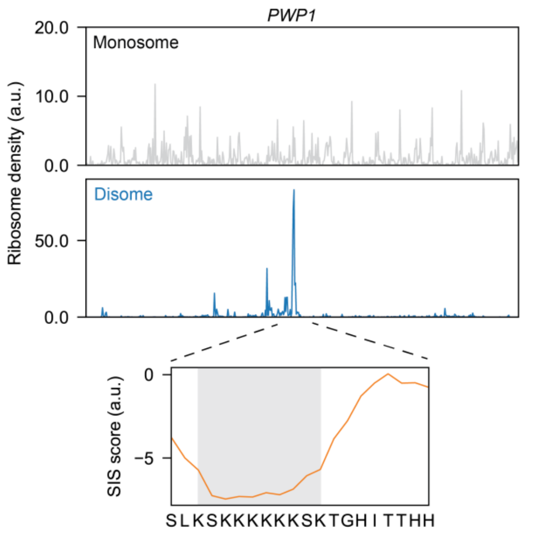

<u>Supplementary Figure 8</u>. SIS analysis identifies consecutive Lys codons as the sequence determinant of disome peaks in a single gene. Monosome (gray, top) and disome (blue, middle) profiles of the *PWP1* gene in WT yeast cells are shown. The SIS profile (bottom) around the disome peaks is shown and the region with consecutive Lys codons is shaded in light gray.

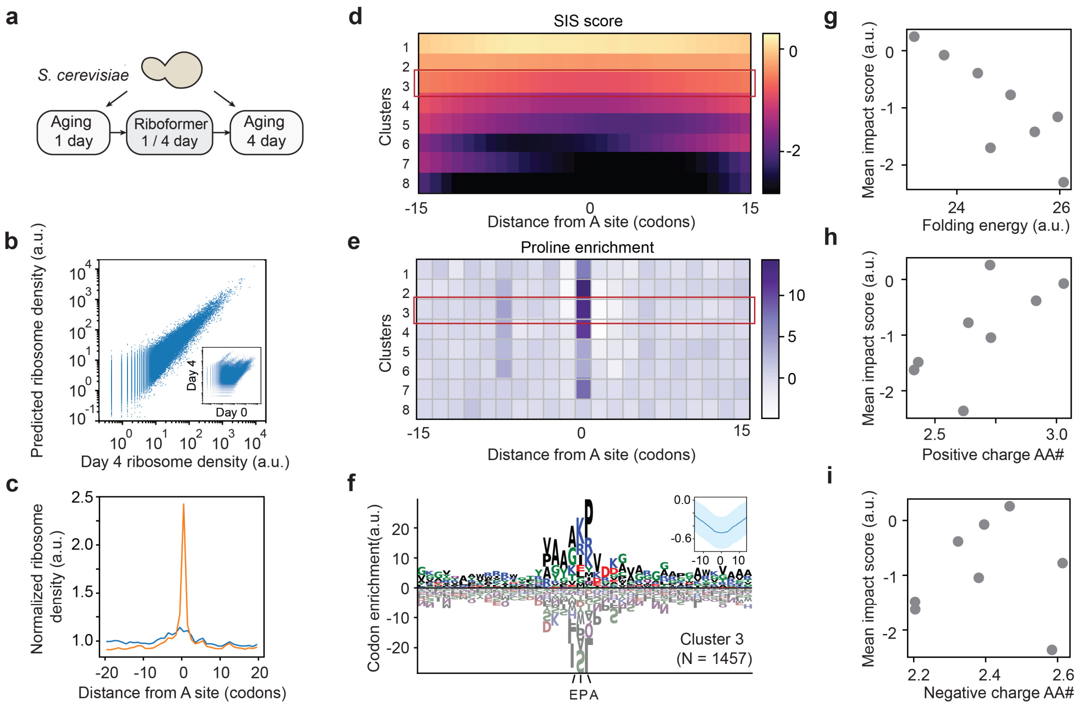

<u>Supplementary Figure 9:</u> Sequence determinant of ribosome pausing in aged yeast. a, ribosome profiles from young and aged yeast were used to train the Riboformer model. **b**, the predicted ribosome density and true ribosome density in the test dataset is shown. Each dot is a single codon of interest, r = 0.94. Inset, comparison of ribosome density in young and old yeast cells, r = 0.44. **c**, the average ribosome occupancy at age-dependent pause sites, n = 6,347. The two ribosome profiles are normalized in the 40-codon window by dividing the mean number of reads across the window. **d**, SIS profiles of all the ribosome stalling sites are grouped into 8 clusters. Cluster 3 is highlighted, which has the lowest SIS in the decoding site. **e**, proline codon enrichment scores for all the clusters (methods). **f**, codon enrichment profile for cluster 3 (n = 1,457). Inset, mean SIS profile for cluster 3. The shaded region represents the standard derivation of all profiles. g-i, comparison of the folding energy of the input sequence, r = -0.84 (**g**), mean number of positive charged amino acids, r = 0.68 (**h**), mean number of negatively charged amino acids, r = 0.05 (**i**) of the input sequence with the mean SIS for 8 clusters. Each dot represents mean values from one cluster.

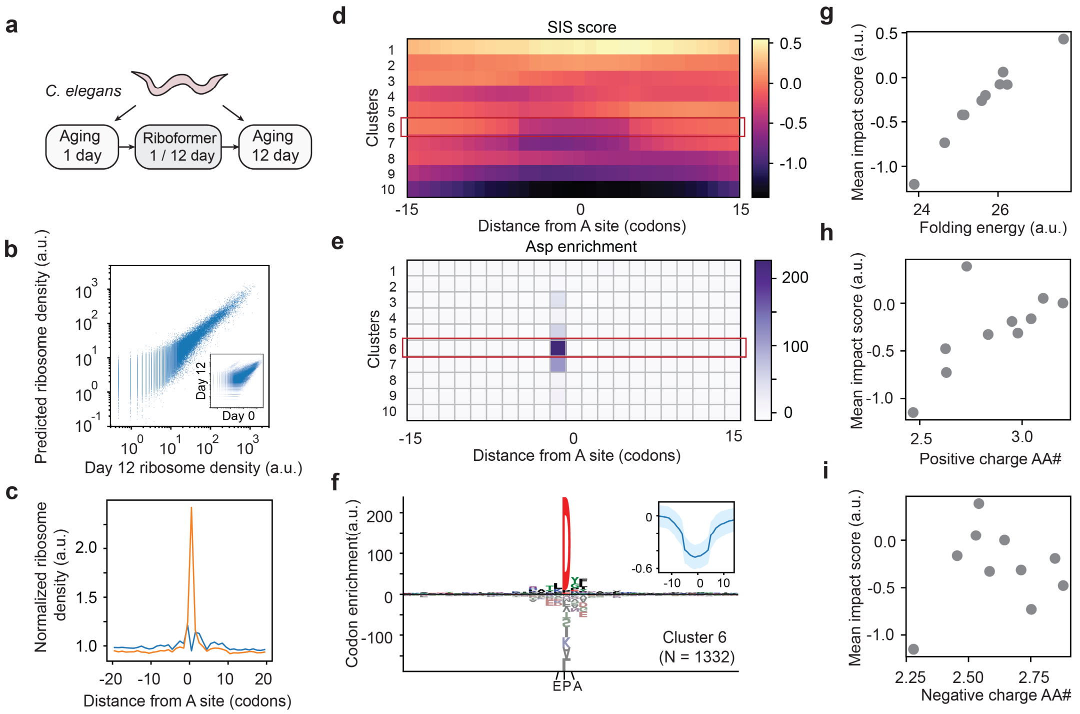

<u>Supplementary Figure 10:</u> Sequence determinant of ribosome pausing in aged worms. a, ribosome profiles from young and aged worm cells were used to train the Riboformer model. **b**, the predicted ribosome density and true ribosome density in the test dataset is shown. Each dot is a single codon, r = 0.91. Inset, comparison of ribosome density in young and old worms, r = 0.81. **c**, the average ribosome occupancy at age-dependent pause sites, n = 8,376. The two ribosome profiles are normalized in the 40-codon window by dividing the mean number of reads across the window. **d**, SIS profiles of all the ribosome stalling sites are grouped into 10 clusters. Cluster 6 is highlighted, which has the lowest SIS in the decoding site. **e**, Asp codon enrichment scores for all the clusters (methods). **f**, codon enrichment profile for cluster 6 (n = 1,332). Inset, mean SIS profile for cluster 6. The shaded region represents the standard derivation of all profiles. g-i, comparison of the folding energy of the input sequence, r = 0.97 (**g**), mean number of positive charged amino acids, r = 0.66 (**h**), mean number of negatively charged amino acids, r = 0.15 (**i**) of the input sequences with the mean SIS for 10 clusters. Each dot represents mean values from one cluster.

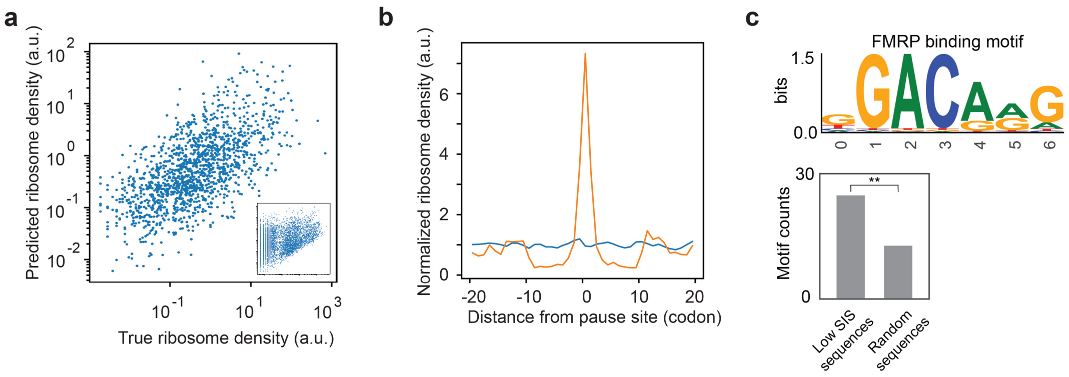

<u>Supplementary Figure 11:</u> Analysis of the ribosome profiles of SARS-CoV-2 canonical open reading frames (ORFs). a, ribosome densities of the SARS-CoV-2 canonical ORFs at 5 and 24 hours post-infection (hpi) in human Vero E6 cells were used to train the Riboformer model. The predicted ribosome density and the true ribosome density in the test dataset at 24 hpi is shown (r = 0.63). Each dot is a single codon of interest. Inset, comparison of ribosome densities at 5 hpi and 24 hpi (r = 0.43). **b,** the average ribosome occupancy of the 24 hpi profiles around the corresponding ribosome stalling sites (orange) and the average ribosome occupancy in the 5 hpi profiles (blue) are shown, n = 170 sites. **c**, upper panel: binding motif of FMRP; lower panel: Comparison of the number of FMRP binding motifs in the sequences with low SIS (below -3, 10 amino acids in length, n = 65) versus the number of FMRP binding motifs in an equal quantity of random sequences with the same length. **p-value = 0.017.

Supplementary Table 1. Prediction performance of Riboformer in terms of the correlation between true and predicted ribosome densities. We performed ten-fold cross-validation tests on four datasets across three species (n = 10). The mean $\pm$ SD of the Pearson correlation coefficients and Spearman correlation coefficients are shown.

Dataset (gene number)	Input data	Output data	Metric	Riboformer
E. coli (1005 genes)	Filtering with the Cm-lysis buffer	Flash-freezing with the high-Mg buffer	Pearson	0.891 $\pm$ 0.002
			Spearman	0.860 $\pm$ 0.003
Yeast (1608 genes)	Monosome	Disome	Pearson	0.763 $\pm$ 0.002
			Spearman	0.620 $\pm$ 0.004
Yeast (2315 genes)	0 day (young)	4 days (old)	Pearson	0.932 $\pm$ 0.001
			Spearman	0.869 $\pm$ 0.002
C. elegans (3030 genes)	1 day (young)	12 days (old)	Pearson	0.920 $\pm$ 0.001
			Spearman	0.827 $\pm$ 0.002

Supplementary Table 2. Prediction performance of Riboformer on lowly expressed genes. We selected a subset of 402 genes in the Mohammad et al. dataset with low ribosome densities (average ribosome density across coding sequence < 0.09). On average 69% of codons in these genes have zero read counts. We performed 3-fold cross-validation tests (n = 3) using the Riboformer model for this dataset. In addition, we have trained the Riboformer model on the high-density genes and tested the model performance on these lowly expressed genes (n = 3). The mean $\pm$ SD of the Pearson correlation coefficients and Spearman correlation coefficients are shown.

Model description	Metric	Riboformer
Three-fold cross-validation on lowly expressed genes (402 genes)	Pearson	0.566 $\pm$ 0.008
	Spearman	0.542 $\pm$ 0.006
Training on highly expressed genes (1005 genes) and testing on lowly expressed genes (402 genes)	Pearson	0.535 $\pm$ 0.003
	Spearman	0.515 $\pm$ 0.003

Supplementary Table 3. Prediction performance of Riboformer for two replicates from the yeast and worm aging datasets. The model performance was evaluated either by using only one of the two replicates, or by taking the average ribosome densities from both replicates. We performed 3-fold cross-validation tests (n = 3), and the Pearson correlation coefficient and Spearman correlation coefficient between the true and predicted ribosome densities are reported (mean $\pm$ SD).

Dataset	Metric	Riboformer		
		Rep1	Rep2	Mean
Yeast	Pearson	0.928 $\pm$ 0.001	0.913 $\pm$ 0.005	0.936 $\pm$ 0.006
(2315 genes)	Spearman	0.808 $\pm$ 0.001	0.816 $\pm$ 0.001	0.862 $\pm$ 0.002
C. elegans	Pearson	0.928 $\pm$ 0.004	0.903 $\pm$ 0.040	0.938 $\pm$ 0.017
(3030 genes)	Spearman	0.732 $\pm$ 0.002	0.765 $\pm$ 0.001	0.822 $\pm$ 0.002

Supplementary Table 4. Comparison of prediction performance of Riboformer with that of different baseline methods in terms of the correlation between true and predicted ribosome densities. Following the original publications, we performed 3-fold cross-validation tests for Riboexp (n = 3), and 10-fold cross-validation tests for RiboMIMO (n = 10). Riboformer model was evaluated in two modes: "seq only mode" which uses the coding sequence as the only input, and the "full mode" which takes two inputs. We used ten-fold cross-validation tests for Riboformer (n = 10). All models were tested on an identical set of genes for each species. The mean $\pm$ SD of the Pearson correlation coefficients and Spearman correlation coefficients are shown.

Dataset (gene numbers)	Metric	Methods			
		Riboexp	RiboMIMO	Riboformer (seq only)	Riboformer (full mode)
E. coli	Pearson	0.642 $\pm$ 0.005	0.657 $\pm$ 0.024	0.848 $\pm$ 0.011	0.891 $\pm$ 0.002
(1005 genes)	Spearman	0.656 $\pm$ 0.002	0.683 $\pm$ 0.013	0.801 $\pm$ 0.015	0.860 $\pm$ 0.003
Yeast	Pearson	0.587 $\pm$ 0.002	0.581 $\pm$ 0.012	0.886 $\pm$ 0.001	0.932 $\pm$ 0.001
(2315 genes)	Spearman	0.489 $\pm$ 0.010	0.491 $\pm$ 0.009	0.798 $\pm$ 0.002	0.869 $\pm$ 0.002
C. elegans	Pearson	0.511 $\pm$ 0.026	0.534 $\pm$ 0.009	0.892 $\pm$ 0.001	0.920 $\pm$ 0.001
(3030 genes)	Spearman	0.467 $\pm$ 0.010	0.497 $\pm$ 0.009	0.784 $\pm$ 0.002	0.827 $\pm$ 0.002

Supplementary Table 5. Prediction performance of Riboformer for correcting experimental bias in yeast. Wu et al. dataset was used to train the Riboformer models. We generated three different datasets: ribosome densities for 21 nt ribosome-protected mRNA fragments (RPFs), 28 nt RPF, and all RPF, under two conditions: lysis buffer with cycloheximide (CHX) and lysis buffer with cycloheximide (CHX)/tigecycline (TIG). Read counts from the CHX condition was used to predict open 40S A sites (21 nt), or occupied 40S A sites (28nt) under CHX/TIG condition. We performed 3-fold cross-validation tests (n = 3), and the mean $\pm$ SD of the Pearson correlation coefficients and Spearman correlation coefficients are shown.

Dataset (Description)	Metric	Riboformer
Wu dataset (CHX 21nt to CHX/TIG 21 nt)	Pearson's	0.731 $\pm$ 0.007
	Spearman's	0.700 $\pm$ 0.002
Wu dataset (CHX all to CHX/TIG 21 nt)	Pearson's	0.680 $\pm$ 0.009
	Spearman's	0.689 $\pm$ 0.005
Wu dataset (CHX all to CHX/TIG 28 nt)	Pearson's	0.798 $\pm$ 0.018
	Spearman's	0.816 $\pm$ 0.001

Supplementary note 1 Relation between the performance of Riboformer and input data characteristics.

Sequence coverage: The averaged ribosome density (RD) can be a metric to evaluate the gene expression levels, while the coverage rate represents the proportion of non-zero counts in the gene coding region. It is worth noting that genes with high averaged ribosome density generally have higher sequencing coverage (Supplementary Figure 2). We separated the genes according to their sequence coverage into 4 groups (from high to low): 80% to 100%, 60% to 80%, 40% to 60% and 20% to 40%, and used 3-fold cross validation to test Riboformer's performance on these 4 gene groups. The reason we chose 3-fold cross-validation is that we observed little difference between the results of 3-fold and 10-fold cross-validation tests in our benchmarking experiments (data not shown). We found that our model performs better for transcripts with high coverage, in accord with previous work[17]. The highly expressed genes usually have high sequence coverage and data quality, which also explains the better prediction accuracy of the Riboformer model. For sequence coverage from 40% to 60%, we achieved a Pearson correlation of 0.62 between true and predicted ribosome densities. Whereas for very poorly expressed transcripts (with more than 60% of zero counts in the coding sequences), we still achieve a Pearson correlation of 0.53 between true and predicted ribosome densities (Supplementary Figure 2).

Model performance on low expression genes: We conducted new experiments to ensure that the prediction performance of our model can be generalized to lowly expressed genes. We trained the Riboformer model using the top 25% of highly expressed genes from the E. coli dataset. The model performance was then tested on 402 lower expressed genes that are not in the training dataset and fall in the 25th to 35th percentile for expression. On average, 69% of codons in these lowly expressed genes have zero read counts. This training and testing process was repeated three times, and we report the average correlation between true and predicted ribosome densities for all codons in the test dataset. In the meantime, we trained and evaluated the baseline models by using only the lowly expressed genes. We found that the model trained on the highly expressed genes generated a Pearson correlation that was only 0.03 less than the baseline models (Supplementary Table 2). This suggests that even when trained on highly expressed genes, the Riboformer model effectively predicts ribosome density for genes with lower expression.

Model performance on replicated data: To evaluate Riboformer's performance on replicated data, we used the two replicates from yeast aging and C. elegans aging dataset[7] for training and testing our models. We trained the Riboformer model using each of the two replicates, as well as the mean

ribosome density from the two replicates. To evaluate model performance, we conducted a 3-fold cross validation test: all the input genes were randomly split in 3 groups. In each fold, we selected one group as the test dataset and the remaining 2 groups as the training dataset. We then calculated the correlation coefficients between predicted and true ribosome densities for the codons in the test datasets. Our analysis revealed that using only one replicate yields high correlations between the true and predicted ribosome densities (Supplementary Table 3). The average Pearson correlation is 0.92 for both yeast and worm. There was a minor increase in correlations (0.02 for both datasets) when using the average ribosome density, suggesting that having more replicates could enhance the signal-to-noise ratio and the model's performance.

Supplementary note 2 Comparison of Riboformer with baseline methods.

Baseline models: RiboMIMO[25] and Riboexp[26] are two state-of-the-art deep learning-based methods to model ribosome distribution based on sequence features. They employed different neural network structures. RiboMIMO uses a multi-input and multi-output (MIMO) architecture. This enables RiboMIMO to predict whole-gene ribosome density distribution from full-length CDS sequences. It utilizes a bidirectional gated recurrent unit (Bi-GRU) network to learn the feature representation for each codon. Riboexp uses a Policy network to select codons that are important for the prediction of ribosome densities. We implemented RiboMIMO and Riboexp based on the source code provided from the original research (https://github.com/tiantz17/RiboMIMO, and https://github.com/Liuxg16/Riboexp). To fairly compare Riboexp and RiboMIMO with our model, we truncated the reference input branch in Riboformer to make it a purely sequence-based model (seq-only mode). We also benchmarked the original design of Riboformer with two inputs (full mode).

Model evaluation: We assessed Riboformer's performance and robustness using a 10-fold cross-validation approach. The training dataset was randomly split into 10 folds. In each fold, one subset of the data was held out as test data while the remaining data were used for training. All the baseline methods were trained following the original publications. More specifically, we used 10-fold cross-validation tests for RiboMIMO and 3-fold cross-validation tests for Riboexp. To evaluate model performance, we used the Pearson and Spearman correlation coefficients to measure the correlations between the predicted and true ribosome densities for all the codons in the test dataset. All models were trained on the same set of highly expressed genes.

Model performance: Our results are reported in Supplementary Table 4 and Supplementary Fig. 3. In our benchmark experiment for the *E. coli* dataset, we have used the top 1005 highly expressed genes. This dataset was also used in the original publications of RiboMIMO and Riboexp. Both methods performed similarly to their original results. The Pearson correlation is 0.66 for RiboMIMO, while the original paper reported a correlation of 0.69. For Riboexp, our benchmark reveals a Pearson correlation of 0.64 between the predicted and true ribosome densities. In the original paper, the Pearson correlation is 0.77 for the top 500 highly expressed genes and 0.61 for the 1375 remaining genes. The reason our correlation was 0.13 lower is that we evaluated the model using a larger set of genes, which included those with low sequence coverage. In contrast, Riboformer's seq-only mode generates a Pearson correlation of 0.81, and the full mode generates a correlation of 0.89.

For the yeast dataset, we selected the 2315 highly expressed genes from the yeast aging dataset (day 4) to benchmark all three methods. For the full mode of Riboformer, we used the yeast aging (day 0) as the reference input. Both RiboMIMO (0.58) and Riboexp (0.59) had slightly lower Pearson correlation coefficients than the *E. coli* dataset. This trend is also consistent with the original publications. For the seq-only mode of the Riboformer model, the average Pearson correlation between the true and predicted ribosome densities is 0.85.

For the *C. elegans* dataset, we selected 3030 highly expressed genes from the aging dataset (day 12) to benchmark all three methods. For the full mode of Riboformer, we used the aging data (day 1) as the reference input. Performance for RiboMIMO and Riboexp on the *C. elegans* dataset was lower compared to the *E. coli* and Yeast datasets, and the Pearson correlations are 0.53 and 0.51 respectively. Riboformer still shows a high correlation between predicted and true ribosome densities (Pearson correlation is 0.88 and 0.94 for the seq-only and full mode).
