# Supplementary material for: Riboformer: a deep learning framework for predicting context-dependent translation dynamics
Source: Nat Commun. 2024 Mar 5;15:2011. doi: 10.1038/s41467-024-46241-8 (PMC10915169; doi:10.1038/s41467-024-46241-8)
Supplement: Supplementary file 3 — Inventory of Supporting Information [file 41467_2024_46241_MOESM3_ESM.docx]

**Supplementary figures**

**Supplementary Figure 1. Prediction performance of Riboformer in relation to the window size of input sequence.**

**Supplementary Figure 2.** **Prediction performance of Riboformer in relation to the sequence coverage of the input data.**

**Supplementary Figure 3. Comparison of the prediction performance of Riboformer with that of different baseline methods across three different species.**

**Supplementary Figure 4: Ribosome pausing in *E. coli* cells with m^1^G37 deficiency.**

**Supplementary Figure 5:**  **Codon level comparison of ribosome density and DMS-seq score.**

**Supplementary Figure 6:**  **Sequence determinants of disome peaks in yeast.**

**Supplementary Figure 7. SIS analysis of disome formation sites.**

**Supplementary Figure 8. SIS analysis identifies consecutive Lys codons as the sequence determinant of disome peaks in a single gene**.

**Supplementary Figure 9: Sequence determinant of ribosome pausing in aged yeast.**

**Supplementary Figure 10: Sequence determinant of ribosome pausing in aged worms.**

**Supplementary Figure 11:**  **Analysis of the ribosome profiles of SARS-CoV-2 canonical open reading frames (ORFs).**

**Supplementary tables**

**Supplementary Table 1. Prediction performance of Riboformer in terms of the correlation between true and predicted ribosome densities.**

**Supplementary Table 2. Prediction performance of Riboformer on lowly expressed genes.**

**Supplementary Table 3. Prediction performance of Riboformer for two replicates from the yeast and worm aging datasets.**

**Supplementary Table 4.** **Comparison of prediction performance of Riboformer with that of different baseline methods in terms of the correlation between true and predicted ribosome densities.**

**Supplementary Table 5.** **Prediction performance of Riboformer for correcting experimental bias in yeast.**

**Supplementary note 1 Relation between the performance of Riboformer and input data characteristics.**

**Supplementary note 2** **Comparison of Riboformer with baseline methods.**
